# Recent Debate over How to Tackle Rapid Increases in Pharmaceutical Expenditure in Japan

**DOI:** 10.31662/jmaj.2019-0047

**Published:** 2020-03-19

**Authors:** Ryu Niki

**Affiliations:** 1 Nihon Fukushi University, Nagoya

**Keywords:** pharmaceutical expenditure, Opdivo rhapsody, cost-effectiveness appraisal

## Abstract

Pharmaceutical expenditure has been rapidly increasing since 2000 in Japan mainly due to successive introduction and diffusion of high-priced new pharmaceuticals (thereafter, drugs). The share of drug expenditure in the national healthcare expenditure rose from 19.6% in 2000 to 22.3% in 2013, a 2.7% point rise in 13 years. In the same period, the share of healthcare personnel's income dropped 3.8% points from 50.2% to 46.4%. Further, in 2016 national healthcare expenditure rose for 3.8%, which is exceptionally high by Japanese standard (roughly 2%), and the main culprit was the rapid increase in drug expenditure due to successive introduction of extremely high-priced drugs.

Due to these changes, drugs have become the main target in current healthcare cost containment policy.

In this article, I briefly explain two debates relating to drug cost and cost control that occurred in 2016 and 2017, respectively, in Japan, based on my two articles that I wrote when I participated in these debates. Although two debates are independent, the first debate that was triggered by an introduction of extraordinary high-priced drug (Opdivo) substantially affected the second debate on how to introduce official cost-effectiveness appraisal of new drugs in Japan.

## 1. Debate over a "Prophecy" That Opdivo Will Destroy Japan

### (1) "Opdivo rhapsody"

In late 2015, Dr. Kunito Hideo, famous oncologist at Red Cross Medical Center, made a sensational "prophecy" that Opdivo (nivolumab) will induce Japan's financial collapse due to its extraordinary high price. The unit price of Opdivo per 100mg was 730,000 yen (about 73,000 US dollars) at that time.

Opdivo is a breakthrough immuno-oncology drug that was originally developed in Japan by Dr. Honjo Tasuku in Kyoto University and was approved for healthcare insurance coverage in 2014 for the first time in the world. At that time, the indication of this drug was limited to melanoma, a very rare skin cancer. However, in 2015, its indication was expanded to lung cancer that is one of the leading causes of death in Japan. Due to this change, potential patients for Opdivo inflated over 100 times, but the extremely high price was preserved.

The annual average cost of Opdivo for lung cancer per patient was as high as 35 million yen (350 thousand dollars) in 2015, and patients must receive Opdivo indefinitely.

Dr. Kunito estimated that the number of lung cancer patients who will annually receive Opdivo will soon reach to 50,000 and that the total annual cost of Opdivo in Japan will reach 1.75 trillion yen (1.75 million dollars) in the near future. Based on this rough estimate, he argued that Opdivo will destroy Japan's financial condition. He even predicted that Japan will become "the second Greece" due to Opdivo and that the national healthcare insurance system will inevitably break up.

In the following year (2016), almost all national newspapers and influential journals cited Dr. Kunito's "prophecy" without criticism. A famous neuroscientist even argued that the total annual expenditure of Opdivo will reach 10 trillion yen if the indication of Opdivo will be further extended in the future. This argument is crazy since this figure is the equivalent to total drug expenditure nowadays.

Further, the Ministry of Finance, which has the strongest authority to make public and social policies among ministries in Japan, used Dr. Kunito's argument as an excuse to narrow the coverage of healthcare insurance.

I called Dr. Kunito's "prophecy" and discourses that were induced by his remarks as "Opdivo rhapsody" and criticized it in 2016.

### (2) My criticism based on theory and historical facts 

I pointed two failures of Dr. Kunito's argument based on health economics and policy.

My first criticism: Dr. Kunito negated the role of the government's healthcare cost containment policy as well as the government's ability to cut the present high price of Opdivo. He then naively extrapolated today's high price of Opdivo as fixed to the future.

However, vast theoretical and empirical researches have revealed that drugs and other technologies are not "independent variables" but are actually "dependent variables" influenced by or amenable to healthcare cost containment policy in countries that have national public healthcare insurance system with price regulation mechanism. (Please note that I use "independent variable" and "dependent variable" as a metaphor and do not use them in strict statistical term.) Historically, there are no nations that bankrupted due to the increase in healthcare cost.

My second criticism: Dr. Kunito does not know the success history of Japan's health care cost containment policy.

In the past, in Japan, there were two cases when rapid healthcare cost increased due to certain disease or when technological advancements were afraid to induce financial collapse of healthcare insurance system: tuberculosis treatment in the 1950s and hemodialysis treatment for renal failure in the 1970s–1980s. The collapse, however, was both prevented due to technology advance-induced price decrease and the government's strict price cutting policy.

As for tuberculosis, total expenditure as well as total number of patients drastically decreased since the 1960s. As for hemodialysis, the annual cost per patient is still as high as 5 million yen (but this figure is nominally half of 10 million yen in 1970), and the number of patients is still increasing (about 330,000 in 2016). However, the share of hemodialysis cost in the national healthcare expenditure remains stable to be under 4.0% for over 20 years. This figure was around 5.0% in 1980, when the number of hemodialysis patients was 36,000, only 10% of today's figure.

Brief Conclusion: Technological advance and the national healthcare insurance system can coexist if appropriate price control and appropriate use of technology are implemented.

### (3) The sequel of the first debate

The Ministry or Health, Labor and Welfare (thereafter, the MHLW) slashed prices of Opdivo and other high-priced drugs including Harvoni (sofosbuvir: antiviral drug in treating hepatitis C) at most by half in 2017 using newly and urgently adopted "special market expansion repricing measure (huge-seller repricing). Due to this measure, without cost-effectiveness appraisal that is described below, the national healthcare expenditure in 2017 decreased compared to 2016 by 0.4% for the first time in 17 years.

## 2. Debate over Cost-effectiveness Appraisal of Drugs with Special Reference to Adjustment Method of New Drug Prices

### (1) Description of policy development in the MHLW

The MHLW set up a "special subcommittee on cost-effectiveness appraisal" in Central Social Insurance Medical Council in 2012. The member of this subcommittee consisted of representatives of medical communities and insurers as well as experts of pharmaceutical economics.

After 5 years of fierce discussion or debate, the subcommittee and the Central Social Medical Council reached to an agreement in principle on October 2017. A new method based on cost-effectiveness appraisal was tentatively implemented in 2018 fiscal year, and the final complete measure was implemented in 2019 fiscal year.

Points of method are as follows:

1. Target of cost-effectiveness appraisal is limited to newly approved drugs or medical devices (thereafter, drugs) that have potentially large market size.

2. The results of cost-effectiveness appraisal are only adopted to decide the reimbursement prices of drugs and not to be used to approve decision of new drugs.

3. Adjustment of prices is based on ICER (incremental cost-effectiveness ratio) of each drug.

4. Basic threshold price is set to be 5 million yen (about 50 thousand USD) per QALY (quality-adjusted life-years).This figure was roughly compatible with NICE's criteria (20,000–30,000 pounds per QALY) in England.

5. Ethical and social factors should be considered on price adjustment including public health factor and certain drugs that have no other alternative therapy.

### (2) My multifaceted evaluation on this method

In 2012, when the subcommittee was set up, I proposed three points that should be noted in cost-effectiveness appraisal (economic evaluation of healthcare), which are as follows:

1. We should not overlook that economic evaluation of healthcare itself incurs substantial cost.

2. There is no "global standard" in economic evaluation.

3. We should not undertake economic evaluation taking the existing extremely high costs of certain drugs for granted.

In the subcommittee, these points were basically accepted, and I support the method abovementioned in principle.

I also judge that the setting of 5 million yen （about 50 thousand USD） as a threshold price in ICER is realistic since it is equivalent to the annual average hemodialysis cost per patient that has already become a de facto threshold for coverage of new technologies.

Nevertheless, I have two objections to this method, which are as follows:

1. The "willingness-to-pay" concept that is considered to be used to decide the threshold of ICER is immature or incomplete and should not to be used. General survey on "willingness to pay" that was once agreed in the subcommittee but then suspended should not be implemented. (Finally, the Central Social Insurance Medical Council decided not to conduct this survey on June 13, 2018.)

2. Although QALY is an academically established concept in economic evaluation of healthcare, it should not be used in actual policy making. Since QALY contains hidden valuation of life, harsh backlash against QALY usage will erupt among disabled persons and sick elderly whose QALY is traditionally evaluated lower than healthy persons.

I recommend to use ICER per life-year saved instead of ICER per QALY for life-saving drugs. The use of ICER per QALY should be limited to drugs that do not increase life-year but to those that improve QOL (quality of life). As for drugs that improve also QOL of patients' family like drugs to treat dementia, this "third-party" effect also should be considered.

#### Brief Conclusion

Based on the actual experiences in other countries, the results of cost-effectiveness appraisal are not consistent and hugely vary and are not a strong tool to decrease drug price or expenditure. It should be mainly used to increase transparency in price decision process as well as preemptively prevent extremely high prices of new drugs.

### (3) The sequel of the second debate

Japanese government decided a wide-ranged "fundamental reform of drug pricing" on December 2017 in order to maintain the national healthcare insurance system as well as to promote technological innovation.

In this reform, the introduction of cost-effectiveness appraisal is a backside measure. The leading measure is direct price cut through strictly limited adoption of "price maintenance premium (innovation premium) or "(special) market expansion repricing (huge-seller repricing)" for new drugs and harsh price cut for long-term listed drugs.

As for the tentative price adjustment based on cost-effectiveness appraisal in 2018 fiscal year, only two drugs (including Opdivo) and one medical device among 17 candidate drugs of medical devices were included, due to large gaps between results of appraisals undertaken by drug companies and those by independent researchers. Unit price of Opdivo was cut by 24% this year following 50% cut in 2017.

## Concluding Remarks

Japan is a definite laggard in healthcare technology assessment. Nevertheless, I hope Japan's recent experiences and debates over how to tackle rapid increase in drug cost may give some suggestions to other countries that are also facing similar problems.

## Article Information

### Conflicts of Interest

None
